# Selbstbestimmung und Motivation bei der Projektarbeit – Eine empirische Analyse zum souveränen Arbeitshandeln

**DOI:** 10.1007/s11612-022-00621-0

**Published:** 2022-01-24

**Authors:** Jörg von Garrel, Ansgar Düben

**Affiliations:** 1 Darmstadt University of Applied Sciences (Hochschule Darmstadt, h_da), Darmstadt, Deutschland; 2 Fraunhofer-Institut für Fabrikbetrieb und -automatisierung, Magdeburg, Deutschland; 3 nexus – Institut für Kooperationsmanagement und interdisziplinäre Forschung, Berlin, Deutschland

**Keywords:** Organisationen im Wandel, Digitale Projektarbeit, New Work, Arbeitswelt der Zukunft, Zukunft der Arbeit, Arbeit 4.0, Home Office, Selbstbestimmung, Arbeitssouveränität, Arbeitszeit, Organisations in Change, Digital Project Work, New Work, Working World of the Future, Future of Work, Work 4.0, Home Office, Self-Determination, Work Sovereignty, Working Time

## Abstract

Dieser Beitrag der Zeitschrift Gruppe. Interaktion. Organisation. (GIO) stellt zentrale Ergebnisse zweier empirischer Studien zur Selbstbestimmung von Arbeit bzw. Arbeitszeit – im Sinne eines arbeits(zeit)souveränen Handelns – vor, die im Auftrag der Deutschen Gesellschaft für Projektmanagement e. V. in den Jahren 2015 und 2020 durchgeführt wurden.

Hintergrund des Beitrages ist der Umstand, dass die fortlaufende digitale Transformation auch die Wissens- und somit die Projektarbeit zunehmend dynamisiert. Arbeitssouveränes Handeln gewinnt dadurch an Bedeutung und stellt neue Anforderungen an die in den Projekten tätigen Wissensarbeiter:innen. Die in diesem Beitrag dargestellten Studienergebnisse beleuchten wesentliche Inhalte zur aktuellen Ausgestaltung der Arbeits(zeit)souveränität, um auf dieser Basis Handlungsempfehlungen für ein motivierendes Arbeitshandeln zu geben. Die Ergebnisse verdeutlichen, dass arbeitssouveränes Handeln unter Mitarbeiter:innen in wissensintensiven Projekten weit verbreitet ist. Gemessen an potenziellen Charakteristika arbeitssouveräner Tätigkeiten (Selbstbestimmtheit, Hierarchiearmut, Zeit- und Ortsunabhängigkeit) arbeiten viele Mitarbeiter:innen „weitgehend“ oder „vollkommen“ arbeitssouverän. In der Selbsteinschätzung ist es ein noch größerer Teil, der die Arbeitstätigkeit als „arbeitssouverän“ einstuft. Diese Tendenz zeichnete sich bereits in der ersten Studie (2015) ab und wird in der aktuellen Untersuchung (2020) bestätigt. Dabei hat die Pandemie-Situation den Grad der Arbeitssouveränität beeinflusst: Die Mitarbeiter:innen arbeiten aktuell orts- und zeitunabhängiger sowie selbstbestimmter als vor der Pandemie. Strukturelle Veränderungen auf Organisationsseite, wie eine Verringerung oder Verflachung von Hierarchien, sind mit diesen Änderungen nicht verbunden.

Auf Ebene der Mitarbeiter:innen wird deutlich, dass soziale Eingebundenheit sowie das Erleben von Kompetenz und Autonomie zentrale Grundbedürfnisse selbstbestimmten Handelns sind, die die Motivation der Mitarbeiter:innen signifikant positiv beeinflussen und in Zusammenhang mit der selbstempfundenen Arbeitssouveränität stehen. Somit bestehen potenzielle Stellschrauben für Organisationen, mit Maßnahmen auf der Strukturebene signifikanten Einfluss auf die Handlungsebene und somit die Motivation der Mitarbeiter:innen zu nehmen.

Souveräner Umgang mit der eigenen Arbeit und Arbeitszeit wird als sinnstiftend sowie als Anerkennung und Ausdruck von Vertrauen gewertet. In der Folge wächst die individuelle Verantwortung der Mitarbeiter:innen, was zugleich größere Handlungsspielräume verspricht. Der Strukturwandel wird von Mitarbeiter:innen insgesamt tendenziell als positiv angesehen (2015 & 2020).

## Abstract (en)

This article from the Journal Gruppe. Interaktion. Organisation (GIO) displays central results of two empirical studies as well as resulting recommendations for action. The subject of those studies was the self-determination of work and working hours—in the sense of work (time) sovereign acting—, which has been conducted on behalf of the Deutsche Gesellschaft für Projektmanagement e.V (German Association of project management e. V.) in the years 2015 and 2020.

Background of the article is the fact, that the continuous digital transformation increasingly changes the dynamic of knowledge-based work, as well as project work. Therefore, work sovereign acting becomes increasingly important and creates new demands from knowledge-based workers. The results of the studies shown in this article illuminate important aspects of the current design of work (time) sovereignty and in conclusion recommend steps for the creation of motivational work measures.

The results of the studies show that sovereignty of work is widespread among employees in knowledge-intensive projects. Measured by potential characteristics of work sovereign activities (self-determination, less hierarchy, independence of time and place), many employees work “largely” or “completely” work-sovereign. In self-assessment it is an even larger proportion that classifies labour activity as “sovereign in work”. This tendency was evident in the first study (2015) and is confirmed in the current study (2020). The pandemic situation has influenced the degree of work sovereignty: employees currently work more independently of time and place and are more self-determined than before the pandemic. Structural changes on the organisational side, such as a reduction or flattening of hierarchies, are not associated with these changes. Results regarding employees show that social integration, the experience of competence and autonomy are central basic needs of self-determined action, which have a significant positive influence on their motivation and are related to self-perceived sovereignty of work. This is therefore an opportunity for organisations to significantly influence the level of action and thus the motivation of employees through measures at the structural level. Sovereign handling with their own work and working time is seen as meaningful as well as a recognition and expression of trust. As a result, the individual responsibility of the employees grows, which promises greater scope for actions. The structural change tends to be seen as positive by employees (2015 & 2020).

## Einführung – Digitalisierung und Arbeitszeitselbstbestimmung

Der wissensintensive Sektor nimmt einen hohen Stellenwert als Innovations- und Beschäftigungsmotor der deutschen Wirtschaft ein (Müller 2014) und er ist bereits heute wie kein anderer Bereich der Wirtschaft durch die Digitalisierung (als technische Innovation) geprägt. (Brockhaus et al. 2020; Köffer und Urbach 2018, S. 18). Zielen die hieraus folgenden (technologischen) Entwicklungen primär auf Effizienzgewinne, werden die Konsequenzen der Digitalisierung für die Arbeitstätigkeiten und -systeme der Mitarbeiter:innen (als soziale Innovation) aber häufig vernachlässigt (Buhr 2014; BMAS 2016). Dabei klingen die Charakteristika einer solchen digitalisierten Wissensarbeit bzw. Digitalarbeit vielversprechend, ermöglichen sie doch häufig ein

1. zeit- und ortsunabhängiges,
2. projektorientiertes (Einmaligkeit/Neuartigkeit der Aufgabe, klares Ziel),
3. wissensbasiertes (hohe Komplexität der Prozesse und Aufgaben, geringe Standardisierung, Problemlösung als Fokus) sowie
4. interaktives und integratives (Kommunikation, Kooperation und Koordination im Unternehmen und mit dem Kunden) sowie
5. vernetztes, globales Arbeiten (vgl. hierzu u. a. Peters et al. 2016a; v. Garrel und Peters 2014; Funken 2016; Widuckel und Molina 2014; Moldaschl und Stehr 2010).

Daraus können für diese „Digitalarbeiter:innen“ zahlreiche Vorteile wie die Flexibilisierung der Arbeit und damit eine bessere Vereinbarkeit von Arbeit und Privatleben, aber auch Risiken aufgrund der Entgrenzung von Arbeit und Privatleben sowie einer ständigen Erreichbarkeit entstehen.

Gegenstand dieses Artikels ist die Vorstellung ausgewählter Ergebnisse von zwei korrespondierenden empirischen Studien zur Arbeits(zeit)souveränität von Mitarbeiter:innen in wissensintensiven Projekten. Die Studien sind im Auftrag der GPM (Deutsche Gesellschaft für Projektmanagement e. V.) in den Jahren 2015 und 2020 durchgeführt worden. Zielte die erste Studie darauf ab, Bedingungen und Optionen von wissensorientierter Projektarbeit sowie resultierende Formen und Grenzen von Arbeitszeit zu analysieren, wurden in der zweiten Studie ausgewählte Fragen erneut aufgegriffen und die Analyse zu einem Ansatz der Arbeitssouveränität erweitert.

Die Ergebnisse ermöglichen eine Darstellung des aktuellen Standes zur Arbeits(zeit)souveränität digitaler Projektarbeit aus Perspektive der Mitarbeiter:innen, auf deren Basis Handlungsempfehlungen und Einschätzungen gegeben werden, wie ein selbstbestimmtes und motivierendes Setting arbeits(zeit)souveränen Handelns ausgestaltet sein kann.

Der Artikel stellt zunächst einen Überblick theoretischer Grundlagen vor, geht dann auf die Methodik und schließlich auf ausgewählte Ergebnisse der genannten Studie ein. Am Ende steht ein Fazit, in dem Handlungsempfehlungen für ein motivierendes und souveränes Arbeitshandeln gegeben werden.

## Theoretische Grundlagen – ein kurzer Diskurs

Der technologische Fortschritt der vierten industriellen Revolution und der damit verbundene Megatrend der Digitalisierung hat als Ergebnis eine anspruchsvolle VUCA-Welt^1^, in der die möglichen Auswirkungen schwer einschätzbar werden: Etablierte Geschäftsmodelle befinden sich in einem labilen, unsicheren und komplexen Marktumfeld, das langfristige Planungen erschwert und schnelle Veränderungsprozesse der Unternehmen erfordert (vgl. Marrold 2018, S. 84). Die Unsicherheit über zukünftige Marktdynamiken und Trends beeinflussen eine genaue Prognose der Erreichung möglicher Unternehmensziele. Die Vernetzung der Arbeits- und Wirtschaftssysteme und die damit verbundene Komplexität erschwert die Entscheidungsfindung, und die Mehrdeutigkeit von Ereignissen und Situationen verhindert die Erstellung genauer Vorhersagen (Dombrowski und Bogs 2020, S. 104). Auf struktureller Ebene der Organisationen als auch auf Handlungsebene der Mitarbeiter:innen sind daher signifikante Änderungen von Arbeitssystemen notwendig, um auch zukünftig innovations- und wettbewerbsfähig zu sein bzw. zu bleiben (Bruckner et al., 2018, S. 2). Strukturell sind Organisationen durch Hierarchien, vertikale Machtstrukturen sowie durch differenzierte Aufgabenteilungen und Delegationen geprägt. Im Gegensatz dazu repräsentieren neue, digitale Organisationen flache Hierarchien, Agilität, Anpassungsfähigkeit, Innovation und eine ausgeprägte Kommunikations- und Kollaborationsfähigkeit (Reinhardt 2020, S. 106). Auf der Handlungsebene verändern neue Technologien die Art und Weise der Arbeit und Kommunikation, Routinetätigkeiten werden automatisiert und von Algorithmen oder Maschinen übernommen. Verstärkte Formen asynchroner Kommunikation der Menschen untereinander fokussieren zunehmen kreative und innovationsträchtige Tätigkeiten. Insgesamt gewinnen somit wissensintensive Tätigkeiten bzw. *Wissensarbeit* (siehe hierzu auch Arlinghaus 2017; Ibert und Kujath 2011; Keil 2007; North 2014; Stiehler und Schabel 2012) an Bedeutung. Zentrale Charakteristika solcher Arbeitstätigkeiten sind:

- Immaterialität der Ergebnisse: Ergebnisse der Arbeitstätigkeiten sind geistiger, unstofflicher und unkörperlicher Natur.
- Integrativität der Prozesse: Der Arbeitsalltag ist nicht standardisierbar. Weiterhin nimmt implizites Wissen in Strukturen, Prozessen und Projekten eine zentrale Rolle für das erfolgreiche Ausführen der Arbeitstätigkeiten ein.
- Umfangreiches Fachwissen: Auf das für die Aufgabenbearbeitung benötigte Wissen lässt sich nicht gebündelt zugreifen und dieses geht über Fachgrenzen hinweg.
- Kontinuierliche Weiterbildung: Notwendige Inhalte von Weiterbildung umfassen Selbstorganisation, Selbstreflektion und einen Arbeitsethos, der über reines „Abarbeiten“ klar definierter Aufgaben hinaus geht.
- Hohe Komplexität der Prozesse und Aufgaben: Wissen muss in Abhängigkeit von Dynamiken in situativen und lokalen Kontexten in Organisationen neu geschaffen, ständig weiterentwickelt und angepasst werden.
- Kundenindividuelle Prozesse und Ergebnisse: Im Kontakt und der Zusammenarbeit mit internen und externen Akteuren sind emotionale und kollaborative Kompetenzen erforderlich (Peters et al. 2016b, 2016a).^2^

Für Organisationen muss es eine zentrale Kernaufgabe sein, die Beschäftigungsfähigkeit der Mitarbeiter:innen bei solchen wissensintensiven Tätigkeiten zu sichern (Burstedde et al. 2021). Dabei sollen Arbeitsbedingungen im Vordergrund stehen, die eine produktive, aber auch nachhaltige und gesundheitsförderliche Arbeitsweise der Beschäftigten fördern (Eichhorst et al. 2015; Eichhorst und Buhlmann 2015). Das Konzept der *Arbeitssouveränität* kann in diesem Kontext ein geeigneter Lösungsweg sein, diese Anforderungen und Bedürfnisse auf Struktur- und Handlungsebene zusammen zu führen. Durch den Begriff der Arbeitssouveränität sollen die Bedarfe (auf der Strukturebene der Organisationen) und Potenziale (auf der Handlungsebene der Wissensarbeiter:innen) zur selbstbestimmten Arbeit zum Ausdruck gebracht werden. Arbeitssouveränität geht über das Management der Arbeitszeit (Zeitunabhängigkeit) und der räumlichen Verortung (Ortsunabhängigkeit) hinaus und fokussiert die Selbstbestimmtheit der Arbeit (Organisation, Prozessgestaltung, Kommunikation, Teamarbeit, Zieldefinition etc.) im Kontext hierarchiearmer Strukturen.^3^ Arbeitszeit im Verständnis der Arbeitssouveränität erstreckt sich als selbstbestimmter Umgang mit Arbeitszeit auf die Dauer, den Umfang und/oder die zeitliche und räumliche (z. B. Home Office) Lage von Arbeit (Peters et al., 2016a, S. 68).

Gerade bei wissensintensiven Tätigkeiten, die durch einen hohen Digitalisierungsgrad geprägt sind (Brockhaus et al. 2020), kann die Möglichkeit zur Arbeitszeitsouveränität zahlreiche Vorteile wie die Flexibilisierung der Arbeit und damit eine bessere Vereinbarkeit von Arbeit und Privatleben, die Reduzierung von Pendelzeiten und die Erhöhung des selbstbestimmten Handelns bieten (Pinnow 2011; Picot und Neuburger 2013). Auf den zweiten Blick wird aber deutlich, dass diesen Chancen auch Risiken (z. B. Entgrenzung von Arbeit und Privatleben, Stress aufgrund zunehmender Verdichtung von Arbeit und ständiger Erreichbarkeit, interne und externe Kommunikations- und Koordinationsaufwände) gegenüber stehen können (Hirsch-Kreinsen 2015), welche neue Herausforderungen mit sich bringen, die u. a. deutlich erhöhte Fähigkeiten der Selbstorganisation, der Kommunikation, Kooperation und Koordination sowie der Komplexitäts- und Problemlösung einfordern (Praeg und Bauer 2017; Abt et al. 2016). Hinsichtlich des Konzeptes der Arbeitszeitsouveränität sei aber betont, dass Organisationen nicht zwangsläufig dieselben Zielsetzungen verfolgen wie Mitarbeiter:innen. So können für Organisationen durch arbeitssouveränes Handeln auch Effekte wie einfachere Erreichbarkeit oder stetige Verfügbarkeit im Vordergrund stehen, so dass u. a. ein steigender Arbeitsumfang bei steigender Arbeitssouveränität in Kauf genommen wird (Ahlers und Lott 2018).

Um Selbstbestimmung und -organisation als Basis arbeitssouveränen Handelns langfristig zu erhalten und innerhalb von Entwicklungen neue Potenziale für die Entfaltung und Bewältigung gegenwärtig nicht vorhersehbarer Anforderungen generieren zu können, können Erkenntnisse aus der Selbstbestimmungstheorie (Deci und Ryan 1985, 2000) nützlich sein. Dieses Konzept besteht aus mehreren Teilkonzepten, wobei für diese Studie insbesondere die Theorie der Basisbedürfnisse (Deci und Ryan 2000) relevant ist. In dieser erläutern Deci und Ryan die Entstehung intrinsischer Motivation. Die in der Selbstbestimmungstheorie konzipierten drei psychologischen Bedürfnisse *Kompetenz, Autonomie und soziale Eingebundenheit* gelten dabei für jeden Menschen gleichermaßen und sind angeboren. Das *Autonomieerleben* erklärt das Bedürfnis eines Individuums, sich selbst im Einklang mit seinen Werten und Interessen als Initiator der eigenen Handlungen zu erleben und über sich selbst zu bestimmen. Das Bedürfnis, sich bei der Zielverfolgung als kompetent und effektiv wahrzunehmen und zu erleben, wird durch den Begriff *Kompetenzerleben* beschrieben. Die *soziale Eingebundenheit* beinhaltet das Bedürfnis des Erlebens sozialer Zugehörigkeit und Verbundenheit mit anderen Personen oder Gruppen. Laut Deci und Ryan stellen die Basisbedürfnisse eine psychologische Notwendigkeit dar und sind somit für das psychische Überleben grundlegend. Die entscheidende Annahme ist, dass die Befriedigung der o. g. Bedürfnisse zu intrinsischer Motivation führt und weiter zu Wohlbefinden und persönlichem Wachstum (Deci und Ryan 2000). Zusammenfassen lässt sich, dass selbstbestimmtes, autonomes Handeln zu einer positiven Stimmung, Selbstachtung, Kreativität, Vertrauen sowie Wohlbefinden führt, wohingegen fremdbestimmte, kontrollierte Handlungen diese möglichen Effekte mindern bis auslöschen.^4^ Die kognitive Evaluationstheorie als weitere Sub-Theorie der Selbstbestimmungstheorie führt die Variabilität im Erleben intrinsischer Motivation weiter aus. Eine Grundannahme dieser Theorie ist, dass externe Faktoren, die die Grundbedürfnisse nach Autonomie, Kompetenz und Zugehörigkeit einschränken, auch die intrinsische Motivation mindern (Deci und Ryan 1985). Damit kann eben auch die Organisation an sich als ein mindernder Faktor intrinsischer Motivation angesehen werden. Intrinsische und extrinsische Motivation können im Arbeitskontext generell selten voneinander getrennt werden. Studien haben gezeigt, dass bestimmte Arbeitsformen durch bestimmte Motivationen begünstigt werden. So unterstützen qualitativ fordernde Aufgaben die intrinsische Motivation und quantitative Aufgaben werden besser bei extrinischer Motivation erledigt (Atabaki und Biemann 2016). Dies zeigt erneut, dass die Steigerung von intrinsischer Motivation durch Arbeitszeitsouveränität nicht grundsätzlich erfolgreich sein muss. Organisationen müssen gleichzeitig aufgrund ihre eigenen Ziele grundsätzlich als extrinsischer Faktor gesehen werden, der zwar die Steigerung intrinsischer Motivation zum Ziel hat, letztendlich diese aber nie selber sein kann (Deci und Ryan 2000).

**Figure 1:**
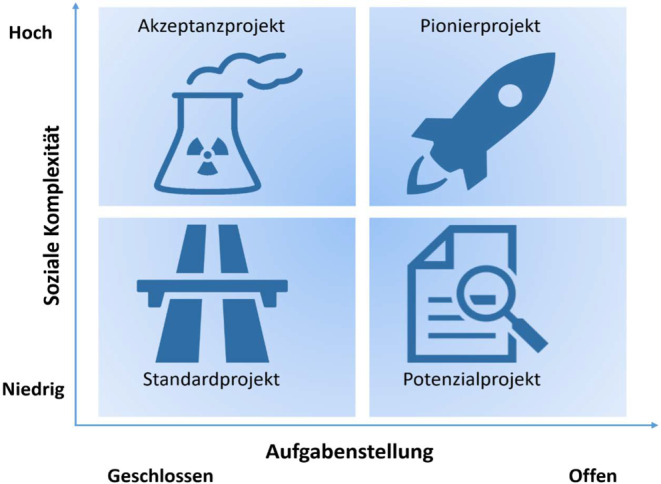

Die Bedarfe und Potenziale arbeitssouverän zu handeln, werden aus arbeitsorganisatorischer Sicht durch die Art der Projektarbeit bzw. deren -komplexität^5^ determiniert. Nach Kuster et al. (2011) können Projekte in vier verschiedene Komplexitätstypen differenziert werden (siehe Abb. 1). Die Projektkomplexität wird mittels der beiden Dimensionen „Soziale Komplexität“ und „Aufgabenstellung“ definiert und spannt somit einen Raum auf, der Projekte nach vier verschiedenen Komplexitätsarten unterscheidet. *Standardprojekte* sind gekennzeichnet durch hohe Erfahrungswerte sowie einfache, standardisierte Abwicklungen. Beispiele hierfür können technische Kundenprojekte sein. *Akzeptanzprojekte* beinhalten eine klare Aufgabenstellung, sind aber häufig aufgrund ihrer hohen sozialen Komplexität mit Akzeptanzproblemen verbunden. Effektive und effiziente Information und Kommunikation sind bei diesen Projektarten zentrale Erfolgsfaktoren. Beispielhaft kann für diesen Typus ein komplexes Informatik-Projekt genannt werden. *Potenzialprojekte* sind häufig mit offenen Fragestellungen verbunden, die mit dem Projektumfeld wenig vernetzt sind und deshalb wenig risikoreich sind. Einfache und kleine Projektorganisation im Sinne von Vorprojekten oder Machbarkeitsstudien sind unter diesem Typ zu subsumieren. *Pionierprojekte* übergreifen mehrere Bereiche, haben hohen Neuigkeitsgehalt und sind risikoreich. Ihr Aufgabenumfang ist schwer abzuschätzen. Die Fusion zweier Firmen kann als Beispiel einer solchen Projektart verstanden werden (Kuster et al. 2011).

## Ergebnisse

### Methode und Stichprobe

Die in diesem Artikel vorgestellten Untersuchungsergebnisse gehen auf zwei quantitative Studien zurück, die im Jahr 2015 sowie 2020 im Auftrag der GPM – Deutsche Gesellschaft für Projektmanagement e. V. durchgeführt wurden. Die Studien weisen inhaltliche Verknüpfungen auf, beleuchten aber jeweils auch singuläre Aspekte. So fokussierte die erste Studie (2015) vor allem den selbstbestimmten Umgang mit Arbeitszeit (Arbeitszeitsouveränität), während die aktuelle Untersuchung (2020) Arbeitssouveränität in verschiedenen Dimensionen betrachtete. Beide Erhebungen erfolgten als Onlinebefragung, die über verschiedene Kanäle beworben wurde, darunter insbesondere Newsletter, die durch die GPM an ihre Mitglieder versandt wurden. Die Zielgruppe bildeten jeweils Mitarbeiter:innen in wissensintensiven Projekten bzw. Wissensarbeiter:innen.^6^

In der Studie von 2015 haben insgesamt 425 Personen, im Jahr 2020 231 Personen teilgenommen. In der Befragung von 2015 waren 43,6 % der Teilnehmenden weiblich und 55 % männlich (1,4 % haben keine Angabe zu ihrer Geschlechtszugehörigkeit gemacht), innerhalb der Stichprobe des Jahres 2020 waren 45 % männlichen und 47 % weiblichen Geschlechts, 1,6 % wählten die Kategorie „divers“ (6,4 % haben keine Angabe zu ihrer Geschlechtszugehörigkeit gemacht). Die Befragten wiesen in beiden Erhebungen ein Durchschnittsalter von ca. 43 Jahren auf.

### Wissens- und Projektarbeit

Die nachfolgende Auswertung zur Beurteilung der ausgeführten Tätigkeiten lässt rückschließen, dass die befragten Projektmitarbeiter:innen Tätigkeiten durchführen, die der Wissensarbeit zugeordnet werden können; ihr Arbeitsalltag ist geprägt von:

- Immaterialität der Ergebnisse ihrer Arbeit (70,0 %) (2015: 55 %)^7^,
- Integrativität der Prozesse (72,9 %) (2015: 73 %),
- umfangreichen Fachwissen (76,5 %) (2015: 89 %),
- kontinuierlicher Weiterbildung (66,2 %) (2015: 62 %),
- hoher Komplexität der Prozesse und Aufgaben (78,4 %) (2015: 83 %) und
- nicht standardisierten, situativen Prozessen und Ergebnissen (68,3 %) (2015^8^: 71 %).

Unterschiede zwischen den beiden Erhebungszeitpunkten in Bezug auf die Tätigkeit bestehen insbesondere hinsichtlich des erhöhten Umfangs der Immaterialität der Ergebnisse (Zustimmung von 55 % auf 70 %) und des verringerten Einsatzes umfangreichen Fachwissens (von 89 % auf 76,5 %). Der Prozess der Digitalisierung scheint hier einerseits eine „Immaterialisierung der Ergebnisse“ voranzubringen, andererseits scheinen auch erste Verschiebungen relevanter Kompetenzanforderungen – wie auch schon durch unterschiedliche Studien bestätigt (u. a. Kirchherr et al. 2018) – deutlich zu werden, sodass Fachkompetenzen an Bedeutung verlieren und Selbst- und Sozialkompetenzen an Bedeutung gewinnen.

**Figure 2:**
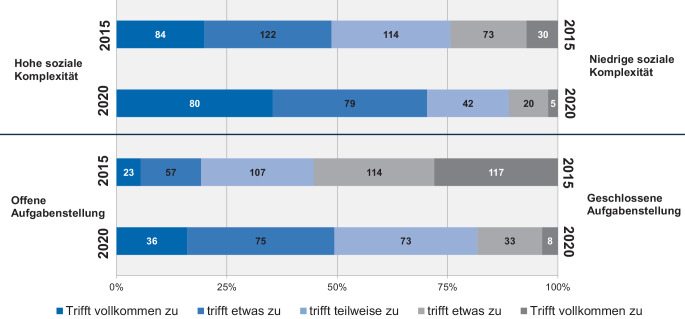

Eng verbunden mit den Charakteristika der Wissensarbeit stellt sich auch die Komplexität der Projekte dar. Orientiert an den von Kuster et al. (2011) konzipierten Dimensionen der „sozialen Komplexität“ und „Offenheit der Aufgabenstellung“ zeigt sich im Zeitvergleich (2015 zu 2020) sowohl eine Zunahme der sozialen Komplexität, als auch der Offenheit der Aufgabenstellung (Abb. 2) und folglich eine Zunahme der Projektkomplexität insgesamt.

Im Jahre 2020 ist bereits über ein Drittel der Befragten (37 %) in Pionierprojekten tätig. Diese zeichnen sich nach Kuster et al. (2011) durch einen hohen sozialen Komplexitätsgrad bei einer zugleich weitgehend offenen Aufgabenstellung aus (Tab. 1). Fast ein Drittel (32,4 %) ist in Akzeptanzprojekten tätig, die durch eine hohe soziale Komplexität, aber eine geschlossene Aufgabenstellung charakterisiert sind. In Standardprojekten mit einem geringeren Komplexitätsgrad (und weitgehend geschlossener Aufgabenstellung) sind 17,4 % der Befragten tätig, weitere 11 % sind in Potenzialprojekten aktiv.

**Table 1:** 

	Anzahl (2020)	Anteil in Prozent (2020)	Anzahl (2015)	Anteil in Prozent (2015)
Akzeptanzprojekte	71	32,4	106	46,5
Standardprojekte	38	17,4	42	18,4
Pionierprojekte	81	37	60	26,3
Potenzialprojekte	24	11	20	8,8
Fehlend	5	2,3	/	/
Gesamt	219	100	228	100

Ein Vergleich mit der Studie von 2015 verdeutlicht dabei, dass die Relevanz an Pionierprojekten signifikant zugenommen hat. Eine hohe soziale Komplexität sowie Offenheit der Aufgabenstellung gewinnt also im Rahmen aktueller Projektarbeiten zunehmend an Bedeutung, was die vorherig benannte Zunahme der Komplexität der Projektarbeit spiegelt.

### Arbeitssouveränität

**Figure 3:**
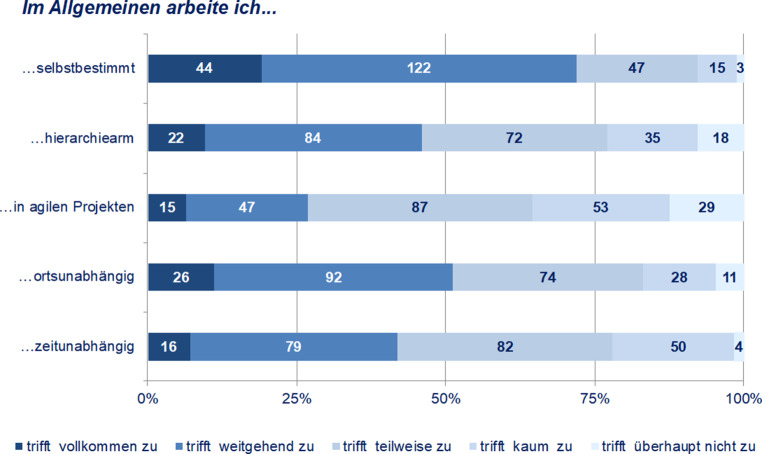

Bei der Betrachtung des Status Quo der einzelnen Faktoren (Selbstbestimmtheit, Hierarchiearmut, Agilität, Ortsunabhängigkeit, Zeitunabhängigkeit) zur Arbeitssouveränität stuft fast Dreiviertel der Befragten (72 %) ihre Arbeitstätigkeit insgesamt als s*elbstbestimmt* ein (Abb. 3). Die Selbstbestimmtheit ist dabei tendenziell durch *zeitunabhängiges* und *ortsunabhängiges Arbeiten* charakterisiert und findet im Kontext *hierarchiearmer Strukturen* statt. Dies gilt für jeweils deutlich mehr als 40 % der Befragten. Somit arbeitet ein Großteil nicht (mehr) in klassischen Zeit- und Raumkonstellationen von Organisationen, wenngleich das Ausmaß selbstbestimmten Arbeitens noch nicht bei allen befragten Mitarbeiter:innen bereits in weitem oder vollem Umfang ausgeprägt scheint. Dies unterstreicht auch das Ergebnis hinsichtlich agiler Arbeitsformen: *Agiles Arbeiten* ist zwar bereits für viele (27 %) Bestandteil der alltäglichen Arbeitsweise, ein ebenso großer Teil (26 %) sieht dies allerdings noch „kaum“ oder „überhaupt nicht“ zutreffend für die Arbeitstätigkeit an. Ein noch größerer Teil der Befragten (37 %) erachtete die Agilität ihrer Arbeit als immerhin „teilweise“ zutreffend. Scheinbar gehört die Organisation von Arbeit in agilen Strukturen und Prozessen für einige Wissensarbeiter:innen bereits zum Alltag, ein größerer Teil digitaler Projektarbeit befindet sich aktuell jedoch noch mitten in einem Erprobungs- bzw. Übergangsprozess.

In einer Reliabilitätsanalyse wurde die Konsistenz der fünf Items unter Berechnung von Cronbach’s Alpha geprüft^9^. Bei Ausschluss des Items „… in agilen Projekten“ lassen sich die Items „selbstbestimmt“, „hierarchiearm“, „ortsunabhängig“ und „zeitunabhängig“ unter Verwendung des arithmetischen Mittelwerts (Mittelwertindex) zu einem einzelnen Faktor „Arbeitssouveränität“ zusammenfügen (vgl. Kuckartz et al. 2013). Der geringe Beitrag des Items „… in agilen Projekten“ zum Gesamtkonstrukt deutet sich bereits in der grafischen Gegenüberstellung (Abb. 3) an. Agile Projektarbeit scheint damit kein die Arbeitssouveränität beschreibendes Charakteristikum zu sein.

**Figure 4:**
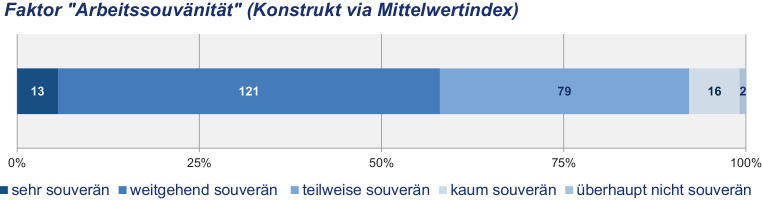

**Figure 5:**
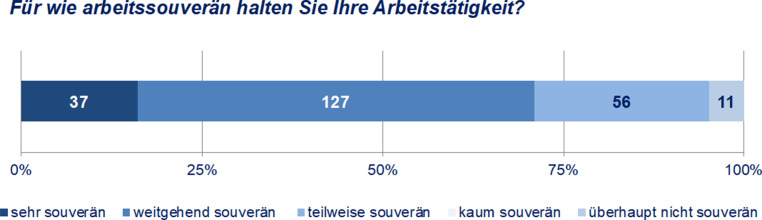

Dem konstruierten Faktor „Arbeitssouveränität“ folgend ist mit 58 % die Mehrheit der Befragten arbeitssouverän tätig (Abb. 4). Dem konstruierten Faktor lässt sich nun die Selbsteinschätzung der Arbeitssouveränität gegenüberstellen (Abb. 5). Dabei zeigt sich, dass mit einem Anteil von 71 % weite Teile der Befragten ihre Arbeitstätigkeit als *arbeitssouverän* einstufen. Das Ausmaß der Arbeitssouveränität wird von den Befragten selbst demnach deutlich höher eingestuft (71 %) als dies durch das theoriegeleitete Konstrukt „Arbeitssouveränität“ (58 %) ausgewiesen wird. Schließlich erreicht mit Ausnahme der Selbstbestimmtheit (72 %) keines der Kriterien eine solch hohe Zustimmung wie die selbstempfundene Arbeitssouveränität.

Eine Korrelationsanalyse unterstreicht den engen Zusammenhang von Arbeitssouveränität und Selbstbestimmung: Die Einschätzung der eigenen Arbeitssouveränität korreliert höchst signifikant mit den einzelnen *Aspekten der Arbeitssouveränität* – mit Ausnahme der agilen Projektarbeit (r_s_ = 0,200, ρ = 0,002). Die höchste Korrelation kann dabei mit der *Selbstbestimmtheit* (r_s_ = 0,487, ρ = 0,000) festgestellt werden, gefolgt von der *Zeitunabhängigkeit* (r_s_ = 0,322, ρ = 0,000), der *Hierarchiearmut* (r_s_ = 0,305, ρ = 0,000) und schließlich der *Ortsunabhängigkeit* (r_s_ = 0,259, ρ = 0,000). Das Verständnis der Befragten hinsichtlich ihrer *Arbeitssouveränität* scheint demnach sehr eng mit der Einschätzung des Ausmaßes der S*elbstbestimmtheit ihrer Arbeit* zusammenzufallen.

Betrachtet man weiterhin, inwiefern die beiden Faktoren der *Wissensarbeit*^10^ und *Arbeitssouveränität* in Verbindung stehen, wird zwischen beiden Faktoren eine signifikante positive Korrelation (r_s_ = 0,193, ρ = 0,005). deutlich. Je intensiver also die Wissensarbeit ausgeprägt ist, desto arbeitssouveräner ist das Arbeitshandeln.

**Figure 6:**
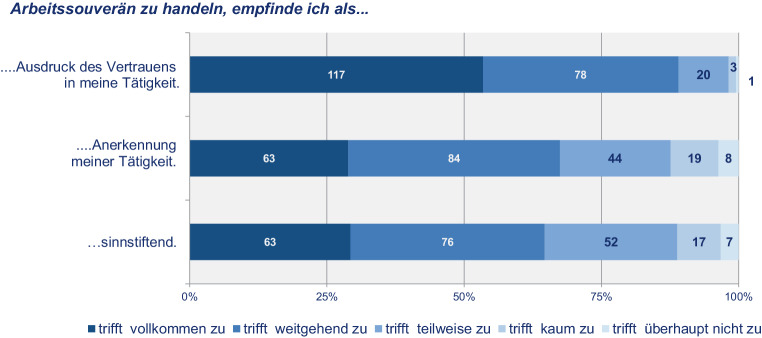

Dabei verbinden die Befragten mit „arbeitssouveränem Handeln“ vor allem einen „Ausdruck des Vertrauens“ in ihre Tätigkeit (89 %), begreifen Arbeitssouveränität zudem aber auch als „sinnstiftend“ (64 %) und als eine Form der „Anerkennung ihrer Tätigkeit“ (64 %) (Abb. 6).

**Figure 7:**
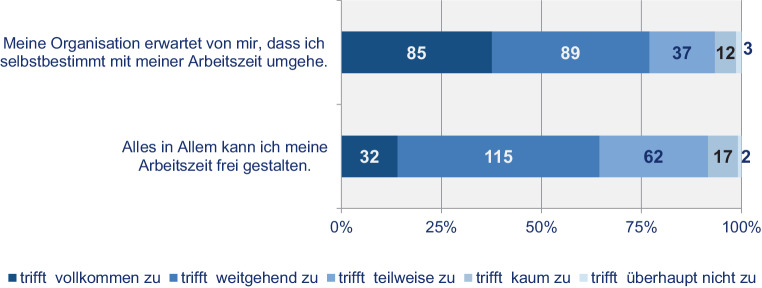

Selbstbestimmtes Arbeiten geht aber offenbar nicht zwingend mit Orts- und Zeitunabhängigkeit einher und muss nicht in jedem Falle in hierarchiearmen Strukturen stattfinden. Arbeitssouveränität bedeutet demnach (u. a.) die grundsätzliche Option, orts- und zeitunabhängig zu arbeiten; nicht immer bzw. nicht alle Mitarbeiter:innen machen davon aber in vollem Umfang Gebrauch. So gaben zusammen 66 % an, die eigene Arbeitszeit *Alles in Allem frei gestalten* zu können (Abb. 7), während das *zeitunabhängige Arbeiten* (s. oben) nur zu 41 % auch genutzt wurde.

Der souveräne Umgang mit der Arbeitszeit kann ein wesentlicher Grundbaustein einer zufriedenstellenden Projektarbeit bilden. Hinzu kommen weitere Ansprüche und Grundbedürfnisse, die in Bezug auf zeitgemäße Arbeitsbedingungen formuliert werden. Grob können diese in die Kategorien des Kompetenzerlebens, sozialer Eingebundenheit und Autonomie differenziert werden. Eine detailliertere Analyse zur Selbstbestimmung und -organisation als Basis arbeitssouveränen Handelns lässt – in Anlehnung an die Erkenntnisse der Selbstbestimmungstheorie (z. B. Deci und Ryan 1985) – alles in allem signifikante Korrelationen zwischen der sozialen Eingebundenheit, dem Autonomieerleben sowie dem Kompetenzerleben eines/einer Wissensarbeiter:in sowie seiner/ihrer intrinsischen Arbeitsmotivation erkennen. Insgesamt ergeben sich folgende statistische Ergebnisse:

Die soziale Eingebundenheit^11^ von Wissensarbeiter:innen korreliert hoch signifikant mit der intrinsischen Arbeitsmotivation^12^ (r = 0,467, *p* = 0,001). Eine Korrelationsanalyse zwischen den Items zur Autonomie^13^ sowie dem Konstrukt der intrinsischen Arbeitsmotivation identifiziert stellenweise hochsignifikante Korrelationen. Konkret ergeben sich folgende Korrelationen:

- Ich habe bei meiner Arbeitstätigkeit das Gefühl, ich selbst sein zu können (r = 0,520, ρ = 0,000).
- Bei meinen Arbeitstätigkeiten muss ich tun, was mir gesagt wird (−)^14^ (r = 0,183, ρ = 0,070).
- Ich bekomme regelmäßiges Feedback zu meiner Arbeit (r = 0,218, ρ = 0,010).

Eine Korrelationsanalyse zwischen den Items zum Faktor Kompetenzerleben^15^ sowie dem Konstrukt zur intrinsischen Arbeitsmotivation identifiziert auch bei diesen Items hochsignifikante Korrelationen zwischen den Items und dem Konstrukt mit geringen bis hohen Zusammenhängen. Konkret ergeben sich folgende Korrelationen:

- An den meisten Tagen fühle ich mich von meiner Arbeitstätigkeit erfüllt (r = 0,646, ρ = 0,000).
- In meinem Job habe ich nicht wirklich die Möglichkeit zu zeigen, wie fähig ich bin (−) (r = 0,459, ρ = 0,000).
- Ich erlebe Situationen, in denen ich mich nicht kompetent empfinde (−) (r = 0,206, ρ = 0,000).

Insgesamt lässt sich also feststellen, dass je sozial eingebundener sowie kompetenter und autonomer sich Wissensarbeiter:innen erleben, desto intrinsisch motivierter sind sie.

### Arbeitssouveränität während der CORONA/COVID 19-Pandemie

Die *CORONA/COVID 19-Pandemie* brachte eine ungewollte Notwendigkeit und Nachfrage nach räumlich und zeitlich flexiblen Arbeitsmöglichkeiten mit sich. Viele der 2020 befragten Projektarbeiter:innen machten von der Option des Arbeitens im Homeoffice Gebrauch, wodurch zugleich die physische Distanz zu möglichen Vorgesetzten mitbedingt wurde. Die Mehrheit (61 %) gab an, dass die CORONA-Lage auch Einfluss auf ihre Arbeitssouveränität genommen habe. Damit war für mehr als die Hälfte aller Befragten (58,4 %) ein *ortsunabhängigeres Arbeiten* die logische Folge. Neben der räumlichen Dimension stuften etwa ein Drittel (32,9 %) ihre Arbeitssituation zudem gegenüber der Zeit vor der COVID-19-Pandemie auch als nunmehr *zeitunabhängiger* ein. Veränderungen der Arbeitsweisen in Zeit oder Raum bedingen einander und bleiben nicht ohne Auswirkungen auf die jeweils andere Arbeitsweise. Demgegenüber sind es nur wenige, die während der CORONA-Situation häufiger agil (4,8 %) als auch hierarchieärmer (4,8 %) arbeiten. Die räumliche Distanz zu Vorgesetzten hat sich offensichtlich nicht allzu stark auf das Hierarchiegefüge bzw. dessen Einflussnahme auf die Arbeitssituation der Projektarbeiter:innen ausgewirkt. 25,5 % der Befragten erachten ihre Arbeitstätigkeit *selbstbestimmter* als vor der Pandemie.

### Der selbstbestimmte Umgang mit Arbeitszeit – und dessen Folgen

Die 2015 durchgeführte Erhebung widmete sich der *Arbeitszeitsouveränität* in besonderem Maße. Ihre Ergebnisse erlauben somit einen tieferen Einblick in die Dimension des souveränen Umgangs mit dem Faktor „Zeit“. Die 2015 befragten Mitarbeiter:innen wissensintensiver Projekte verstanden unter einem *selbstbestimmten Umgang mit Arbeitszeit* („Arbeitszeitsouveränität“) mehrheitlich die Verteilung, d. h. Beginn und Ende (Uhrzeit) des Arbeitstags selbst zu bestimmen (92 %), Arbeitsaufgaben selbstbestimmt durchführen zu können (87 %), die Arbeit im Homeoffice (83,5 %) und die Möglichkeit, Überstunden abbauen zu können (72 %).

Die Verfügungsgewalt hinsichtlich der Verteilung und der räumlichen Verortung spielt demnach eine sichtlich wichtigere Rolle als der Volumenaspekt, d. h. die Handhabe, nach eigenem Ermessen Arbeitszeitkontingente zunächst als Mehrstunden aufzuwenden, um diese dann zu einem späteren Zeitpunkt als Freizeit wieder zur Verfügung zu haben. So wich (2015) z. B. auch das im Durchschnitt an sich gewünschte (wöchentliche) Arbeitszeitvolumen von 36,9 h deutlich von dem tatsächlich geleisteten (Durchschnitts‑)Wert von 44,1 h ab. Und etwa 60 % der Befragten führten aus, sie würden als Folge ihrer Arbeitssouveränität mehr/länger arbeiten; nur 11,5 % gaben an, deutlich weniger/kürzer zu arbeiten.

Und doch ist der selbstbestimmte Umgang mit Arbeitszeit von den Befragten mehrheitlich positiv konnotiert: Der überwiegende Teil der befragten Projektmitarbeiter:innen begriff (2015) Arbeitszeitsouveränität überwiegend als *Freiheit* (85 %) und als *effizient* (75 %) sowie allgemein als *Chance* (73 %) und war der Auffassung, Arbeitszeitsouveränität trage zur persönlichen *Lebensqualität* bei (86 %). Sie eröffne den Projektbeteiligten größere Handlungsspielräume (80 %), ermögliche die Balance von Berufs- und Familienleben (72 %) und fördere die Produktivität der Projektakteure (70 %).

Zu beiden Befragungszeitpunkten erklärte jeweils eine deutliche Mehrheit der Befragten, *ihre Organisation erwarte*, dass sie selbstbestimmt mit ihrer Arbeitszeit umgingen. Im Jahr 2015 waren es 72,5 % und in der Befragung von 2020 mit 79 % sogar noch etwas mehr (Abb. 7). In der Studie von 2015 gaben die meisten Befragten hierzu an, es sei der jeweiligen Organisation *egal, wie lange (Stunden/Tag)* und *wann (Uhrzeit Beginn/Ende)* gearbeitet und dass die Arbeitszeit ohnehin bei mehr als der Hälfte (58 %) nicht kontrolliert werde, da zuvorderst die *termingerechte Leistungserbringung von Relevanz* sei. Somit kommt hinsichtlich des Zeitmanagements insbesondere der individuellen Eigenverantwortung der höchste Stellenwert zu, während der Einfluss der Organisation auf die eigene Arbeitszeit vergleichsweise gering eingestuft wird: 83 % der Befragten (2015) sahen die Projekte und somit die individuellen Herausforderungen und Situationen für die Arbeitszeit maßgebend. Arbeitsverträge oder Vorgaben der Linie bzw. Organisation (jeweils ca. 41 %) scheinen demgegenüber deutlich zurückzustehen. Ein wesentlicher, die Arbeitszeit bestimmender Faktor (71,4 %) ist die eigene Fähigkeit die Arbeitszeit zu managen.

Als *Folge selbstbestimmten Umgangs mit Arbeitszeit* sieht die deutliche Mehrheit der Befragten (90,4 %) ein Anwachsen ihrer *Verantwortung* als Mitarbeiter:in, die von einem Großteil (80,5 %) zugleich aber auch insgesamt als Eröffnung größerer *Handlungsspielräume* eingeschätzt wird (2020). Nahezu identische Werte konnten bereits in der Untersuchung von 2015 ermittelt werden: Hier wiesen 92 % der Befragten eine steigende Verantwortung und 80 % größere Handlungsspielräume als Ergebnis ihrer Arbeitszeitsouveränität aus. Arbeitszeitselbstbestimmung wird von Organisationen zunehmend erwartet (s. oben). Somit verlagern sich hier auch verschiedene Aufgaben und Verantwortungen und damit auch Kompetenzanforderungen in Richtung der Mitarbeiter:innen. So waren 60 % der Befragten der Auffassung, dass Arbeitszeitselbstbestimmung dazu führe, dass die Ansprüche der Mitarbeiter:innen an sich selbst steigen (2015).

Die Organisation scheint als Akteur in den Hintergrund zu treten. Faktisch setzt diese aber durch ihre Erwartung an eine termingerechte Erbringung einer einwandfreien Arbeitsleistung den Zielmaßstab und damit schließlich eine, den gesamten Arbeitsprozess umspannende extrinsische Motivation. Die Druckwirkung dieses Motivators auf die Leistungsansprüche an sich selbst führt – bei aller positiven Konnotation (s. oben) – durchaus zu Negativfolgen, die auch die Kehrseite der Arbeitszeitsouveränität offenlegen: So zeigte die Untersuchung von 2015, dass als Folge von Arbeitszeitsouveränität tendenziell eine Zunahme von Zeit- und Erfolgsdruck, wie auch ein steigendes Risiko zum Burnout gesehen wird und diese zu Mehrarbeit und längeren Arbeitszeiten (nach 18:00 Uhr und am Wochenende) führe.

Es zeigt sich aber auch, dass eine Arbeitszeitselbstbestimmung dem Selbstbewusstsein und Selbstverständnis von Mitarbeiter:innen entspricht und (z. B. gegenüber der Organisation) eingefordert wird: In der Untersuchung von 2015 zeigten sich 23,7 % derjenigen, die sich noch nicht als Arbeitszeitsouveränen ansahen, bereit *„… nötigenfalls auch den Arbeitgeber (Organisation) zu wechseln“*, wenn sie infolgedessen mehr Freiheiten im Umgang mit der Arbeitszeit erlangen könnten.

**Figure 8:**
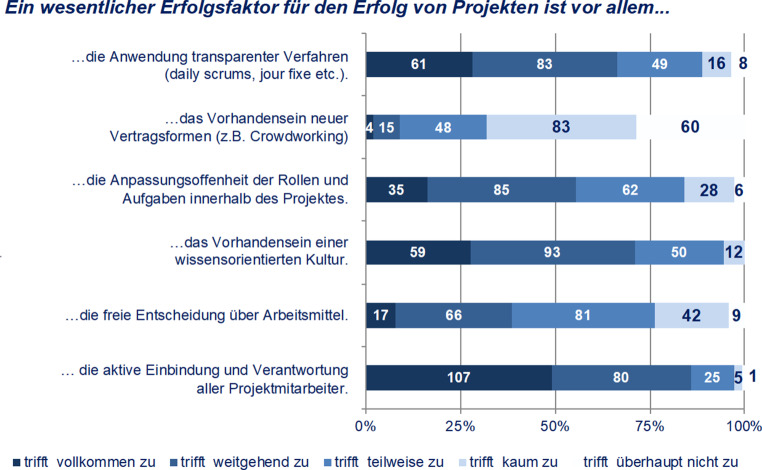

Offensichtlich weisen Mitarbeiter:innen wissensintensiver Projekte nicht nur ein wachsendes Selbstwirksamkeitsbewusstsein, sondern richten im Zuge des wachsenden Selbstbewusstseins auch vermehrt Forderungen an ihre Organisation. So wird der Erfolg von Projekten auch von Rahmenbedingungen abhängig gemacht, die im Verantwortungsbereich der Organisation liegen: Die Befragten gaben mehrheitlich an, dass ihnen für die erfolgreiche Durchführung von Projekten transparente Verfahren, Anpassungsoffenheit (bzgl. Rollen und Aufgaben) und Wissenskultur geboten werden müssen, sowie dass sie selbst in die Verantwortlichkeit aktiv eingebunden werden müssen. Demgegenüber spielen Freiheiten bezüglich der Wahl der Arbeitsmittel sowie neue Vertragsformen eine vergleichsweise untergeordnete Rolle (Abb. 8).

## Fazit und Ausblick

Die hier dargestellten Studien analysieren den selbstbestimmten Umgang mit Arbeitszeit (Arbeitszeitsouveränität) (2015) sowie der arbeitssouveränen Gestaltung (2020) zwischen Organisationen und Wissensarbeiter:innen. Sie verdeutlichen, dass die (arbeits-)organisatorische Komplexität der Projektarbeit von Wissensarbeiter:innen zugenommen hat. Gewachsen ist sowohl die soziale Komplexität als auch die Offenheit der Aufgabenstellung. In diesem Kontext verlieren „klassische Fachkompetenzen“ an Bedeutung und es gewinnen Selbst- und Personalkompetenzen an Relevanz. Arbeitssouveränes Handeln wird dabei sowohl vonseiten der Organisation erwartet als auch durch die Wissensarbeiter:innen genutzt. Dennoch stehen in diesem Konstrukt für die Wissensarbeiter:innen insbesondere selbstbestimmtes und -organisiertes Handeln – und weniger eine örtliche und räumliche Flexibilität – im Vordergrund. Dabei verdeutlichen die Ergebnisse, dass je wissensintensiver die Tätigkeiten sind, desto arbeitssouveräner wird gehandelt.

Selbstbestimmtes Handeln nimmt offenbar positiven Einfluss auf die Arbeitsmotivation und ist dabei zentral daran gebunden, wie (1) kompetent das Handeln erlebt wird, (2) autonom gehandelt werden kann und (3) der/die Akteur:in sozial eingebunden ist. Organisationen auf der Strukturebene und Wissensarbeiter:innen auf der Handlungsebene sollten also diese drei „Grundbedürfnisse“ der Selbstbestimmungstheorie auch in einer arbeitssouveränen (Arbeits‑)Welt systematisch gestalten. Dabei ist insbesondere das Autonomieerleben bei arbeitssouveränem (Arbeits‑)Handeln noch weiter zu untersuchen, da dieses Konstrukt noch nicht klar herausgearbeitet werden konnte. Dies verwundert aber nicht, denn das Bedürfnis nach Kompetenz fokussiert darauf, als wichtig erachtete Ziele zu erreichen und dadurch erwünschte Ergebnisse zu erzielen. Dieser Wunsch deckt sich häufig mit einem – der Autonomie (im ersten Moment) entgegenstehenden Effekt der Anerkennung oder des Feedbacks von bzw. durch Vorgesetzte oder Kolleg:innen.

Basis für ein arbeitssouveränes Handeln ist, dass die notwendige technische, aber auch strukturelle Infrastruktur geschaffen wird (u. a. Bereitstellung der notwendigen Hard- und Software, Zugriffe zu Systemen und IT-Tools), aber natürlich auch räumliche Kapazitäten und zeitliche Strukturen (Festlegung und Einhaltung von Arbeitszeiten und -aufträgen) berücksichtigt werden und in eine individuelle Ausgestaltung der Arbeitssouveränität Eingang finden. Die Studien verdeutlichen, es existiert nicht „die“ Arbeitssouveränität, sondern es bedarf individueller Aushandlungs- sowie Gestaltungsprozesse. Zentral ist hierbei auch die Kommunikation über die Ausgestaltung des Arbeitens im Homeoffice: Die Führungskraft vermittelt die Rahmenbedingungen und gegenseitige Erwartungen werden geklärt. Regelmäßige Termine zur Evaluation der Zusammenarbeit bzw. für Rückmeldungen und Feedbacks über räumliche Distanz werden vereinbart. Dabei sollte die Selbstführung der Mitarbeitenden gefördert und Raum für Entwicklungen geschaffen werden. Mitarbeitergespräche und Zielvereinbarungen nehmen in diesem Kontext eine zentrale Bedeutung ein, um Arbeitssouveränität auf Struktur (Organisation) und Handlungsebene (Mitarbeiter:in) zu verbinden.

Eine Möglichkeit, Autonomie- und Kompetenzerleben zu erhöhen, kann durch eine Verlagerung operativer Entscheidungskompetenzen zur/m Wissensarbeiter:in geschehen. Dadurch können Wissensarbeiter:innen eigenständig über die Gestaltung und Vorgehensweise ihrer Arbeit entscheiden.

Wenn auf Formen der Zeiterfassung verzichtet wird, besteht die Gefahr, dass der Vorteil, größere Freiheit bei der Zeitgestaltung zu haben, in Verbindung mit der Erwartung, ständig erreichbar zu sein, auch zu einem höheren Arbeits- und Zeitdruck führen kann. Denn, wenn nicht mehr die Arbeitszeit, sondern Zielvereinbarungen, Deadlines und Projekttermine die Arbeit determinieren, kann die Verweigerung oder Einschränkung der Kommunikationsmöglichkeit den Druck erhöhen und Stress verursachen.

Soziale Ressourcen, wie Unterstützung durch Kolleg:innen aber auch Anerkennung vom Chef, individuelle Förderung, positives Feedback von Kund:innen und vieles mehr sind Faktoren, die eine soziale Eingebundenheit auch bei arbeitssouveränen Tätigkeiten ermöglichen. Zentral ist in diesem Kontext auch der Aus- bzw. Aufbau einer entsprechenden Unternehmenskultur, in der Unterstützung als auch ein unternehmensweiter Offline- und Online-Austausch möglich ist. Neben dem „Vorleben“ einer solchen Kultur durch das Management kann auch das Wertschätzen der Kompetenzen und deren Beiträge zum Unternehmenserfolg das Kompetenzerleben fördern.

Für Mitarbeitende, die in einer (möglicherweise aktuell durch die *COVID 19-Pandemie* erzwungenen) Arbeitssouveränität aktuell eher Nach- denn Vorteile sehen, können explizite Vereinbarungen förderlich sein. Festgelegte Regeln, die bspw. unbezahlte Überstunden vermeiden als auch klare Arbeitsanweisungen beinhalten, können in einer „flexiblen Arbeitswelt“ Struktur- und Stabilitätsanker sein.

## Footnotes

1 **v**olatility (~ Unbeständigkeit), **u**ncertainty (Unsicherheit), **c**omplexity (Komplexität), **a**mbiguity (Mehrdeutigkeit).

2 *Wissensarbeit* wird empirisch über die sechs dargestellten Charakteristika und deren Ausprägung im jeweiligen Arbeitskontext als latentes Konstrukt gemessen.

3 *Arbeitssouveränität* wird im Rahmen der Studie als latentes Konstrukt über die zentralen Eigenschaften der Selbstbestimmtheit, Hierarchiearmut, Ortsunabhängigkeit, Zeitunabhängigkeit sowie agilen Projektarbeit gemessen.

4 Empirisch kann die Theorie mittels Korrelationsanalysen zwischen den Grundbedürfnissen sowie der Motivationslage gemessen werden. Detailliertere Ausführungen zur Operationalisierung der Selbstbestimmungstheorie sowie ihrer grundlegenden und relevanten Konzepte mit einem besonderen Fokus auf Wissensarbeit(er*innen) findet sich u. a. bei Thürmer (2019).

5 Der Fragebogen erfasst die Komplexität dabei über ein semantisches Differential, das explizit die „Offenheit der Aufgabenstellung“ und die „soziale Komplexität“ abfragt.

6 Die Zielgruppenerreichung wurde in verschiedenen Dimensionen mittels spezifischer Fragen zur Projektarbeit und zur Wissensarbeit, wie zur Organisationsgröße, Beschäftigungsdauer, Vertragsbasis und Position in Organisation und Projekt geprüft und sichergestellt.

7 Hierzu erfolgte eine Dichotomisierung einer 5‑stufigen Likert-Skala. Die Skalenniveaus „Trifft vollkommen zu“ und „trifft weitgehend zu“ wurden zu „trifft zu“ zusammengefasst, die übrigen drei Niveaus zu „trifft nicht zu“.

8 2015 war das Item als „Kundenindividuelle Prozesse und Ergebnisse“ formuliert.

9 Unter Einbezug aller fünf Items ergibt sich ein α von 0,682. Dieser Wert liegt unterhalb der allgemeinhin als Faustregel angesetzten Wert von 0,7 (vgl. Kuckartz) für ein als „akzeptabel“ anzusehendes Konstrukt. Durch Eliminierung des Items „… in agilen Projekten“ lässt sich der Wert auf α = 0,710 erhöhen.

10 Eine Reliabilitätsanalyse ergibt für den Faktor „Wissensarbeit“, der aus den sechs Merkmalen (vgl. Kapitel 2 „Theoretische Grundlagen“) gebildet wird, mit einem Cronbach’s Alpha von 0,704 ein akzeptables Konstrukt.

11 Dieses Konstrukt wurde aus 3 Items gebildet („Ich mag die Menschen, mit denen ich zusammenarbeite.“, „Ich fühle mich bei der Arbeit sozial eingebunden.“ sowie „Ich habe den Eindruck, dass meine Arbeit anerkannt wird.“) und kann mit einem Cronbach’s Alpha von 0,703 als reliables Konstrukt genutzt werden.

12 Dieses Konstrukt wurde aus 2 Items gebildet („Ich strenge mich bei der Arbeit an, weil ich Spaß an meiner Arbeit habe.“ sowie „Ich strenge mich bei der Arbeit an, weil die Tätigkeit, die ich leiste, interessant ist.“). Mit einem Spearman-Brown Wert von 0,867 kann auch hier ein Faktor gebildet werden.

13 Die Bildung eines reliablen Konstruktes ist aufgrund eines Cronbach’s Alpha von 0,422 nicht möglich.

14 Items mit diesem Zeichen sind negativ formuliert und wurden daher bei den statistischen Auswertungen umgepolt.

15 Die Bildung eines reliablen Konstruktes ist aufgrund eines Cronbach’s Alpha von 0,575 nicht möglich.
